# Massively Multiplayer Online Games and Well-Being: A Systematic Literature Review

**DOI:** 10.3389/fpsyg.2021.698799

**Published:** 2021-06-30

**Authors:** Lisa Raith, Julie Bignill, Vasileios Stavropoulos, Prudence Millear, Andrew Allen, Helen M. Stallman, Jonathan Mason, Tamara De Regt, Andrew Wood, Lee Kannis-Dymand

**Affiliations:** ^1^School of Health and Behavioural Sciences, University of the Sunshine Coast, Maroochydore, QLD, Australia; ^2^Institute of Health and Sports, Victoria University, Melbourne, VIC, Australia; ^3^School of Psychology, National and Kapodistrian University of Athens, Athens, Greece; ^4^Thompson Institute, University of the Sunshine Coast, Maroochydore, QLD, Australia

**Keywords:** MMOs, internet gaming, systematic literature review, PRISMA, well-being, massively multiplayer online

## Abstract

**Background:** Massively multiplayer online games (MMOs) evolve online, whilst engaging large numbers of participants who play concurrently. Their online socialization component is a primary reason for their high popularity. Interestingly, the adverse effects of MMOs have attracted significant attention compared to their potential benefits.

**Methods:** To address this deficit, employing PRISMA guidelines, this systematic review aimed to summarize empirical evidence regarding a range of interpersonal and intrapersonal MMO well-being outcomes for those older than 13.

**Results:** Three databases identified 18 relevant English language studies, 13 quantitative, 4 qualitative and 1 mixed method published between January 2012 and August 2020. A narrative synthesis methodology was employed, whilst validated tools appraised risk of bias and study quality.

**Conclusions:** A significant positive relationship between playing MMOs and social well-being was concluded, irrespective of one's age and/or their casual or immersed gaming patterns. This finding should be considered in the light of the limited: (a) game platforms investigated; (b) well-being constructs identified; and (c) research quality (i.e., modest). Nonetheless, conclusions are of relevance for game developers and health professionals, who should be cognizant of the significant MMOs-well-being association(s). Future research should focus on broadening the well-being constructs investigated, whilst enhancing the applied methodologies.

## Introduction

Internet gaming is a popular activity enjoyed by people around the globe, and across ages and gender (Internet World Stats, 2020). With the addition of Internet Gaming Disorder (IGD) in the 5th edition of the Diagnostic and Statistical Manual for Mental Health Disorders (DSM-5; American Psychiatric Association, 2013) as a condition requiring further study, followed by the introduction of Gaming Disorder (GD) as a formal diagnostic classification in the 11th edition of the International Classification of Diseases (ICD-11; World Health Organization, 2019), research concerning the associated adverse effects of gaming has increased (Kircaburun et al., 2020; Teng et al., 2020). Accordingly, a series of potentially harmful aspects of internet gaming, such as reduced social skills, aggression, reduced family connection, interruptions to one's work and education have been cited (Pontes et al., 2020).

Despite such likely aversive connotations, the uptake of internet gaming continues to increase. Recent statistics suggest that 64% of adults in the United States (U.S.) are gamers, 59% of those being male, with the average age range situated between 34 to 45 (Entertainment Software Association, 2020). Of note is that 65% of those gamers are playing with others online or in person and they spend an average of 6.6 h playing per week with others online. Similarly, a survey of 801 New Zealand households (2,225 individuals) revealed that two-thirds play video games, with 34 years being the average age (Brand et al., 2019).

Such high levels of game involvement have been interwoven with high reports of potential well-being benefits in the U.S. sample, including 80% for mental stimulation, 63% for problem solving, 55% for connecting with friends, 79% for relaxation and stress relief, 57% for enjoyment, and 50% for accommodating family quality time (Entertainment Software Association, 2020). Interestingly, 30% of U.S. gamers met a good friend, spouse, or significant other through gaming (Entertainment Software Association, 2020). Thus, video gaming does offer benefits, especially for one's socialization; indeed, gaming can simultaneously engage multiple online players (Pierre-Louis, 2020; Pontes et al., 2020).

Multiplayer online games involve a broad genre of internet games, which entail participants playing with others in teams or competing within online virtual worlds (Barnett and Coulson, 2010). A 2017 report of 1,234 Australian households (3,135 individuals) found 67% regularly played video games on computers, tablets, mobile phones, handheld devices, and gaming consoles, with 92% of those playing online with others (Brand et al., 2017). When the “multiple-players” component allows the concurrent inclusion of large numbers (i.e., masses) of gamers, games are referred as massively multiplayer online games (MMOs; Stavropoulos et al., 2019). Such games employ the internet to simultaneously host millions of users globally. Participants tend to be organized in groups/teams/alliances competing with each other in the context of game worlds with progressively higher demands and challenges (Adams et al., 2019). Massively multiplayer online role-playing games (MMORPG) expand on this format of play with the introduction of role-playing characteristics through the creation of an avatar. This involves the player establishing their own customizable character for their gameplay, providing an opportunity for gamers to experiment with their own identity in a safe environment (Stavropoulos et al., 2020). Thus, MMORPGs constitute a distinct subgenre of MMOs.

A preponderance of recent research on MMOs has focused specifically on the negative effects of problematic gaming or IGD (Kircaburun et al., 2020; Pontes et al., 2020). For instance, a systematic review conducted by Männikkö et al. (2017) focused on health-related outcomes of problematic gaming behavior. This review aligns with prior research that looked at the risk factors and adverse health outcomes of excessive internet usage, particularly among adolescents (Lam, 2014; Goh et al., 2019). Despite these efforts, Sublette and Mullan (2012) suggested that the evidence regarding the negative health consequences of gaming is inconclusive (e.g., overall health, sleep, aggression). As Internet games, and especially MMOs, may be also played moderately, they can accommodate a series of beneficial effects for the users such as socialization, a sense of achievement, and positive emotion (Halbrook et al., 2019; Zhonggen, 2019; Colder Carras et al., 2020). Accordingly, the systematic literature review of Scott and Porter-Armstrong (2013) aimed to offer a more balanced view of the whole range of the positive and the negative effects of participation in MMORPGs, including on the psychosocial well-being of adolescents and young adults. They studied six research articles, where both negative and positive outcomes were identified; for instance, they concluded that problematic/pathological gaming associated with the negative outcomes such as depression, disrupted sleep, and avoidance of unpleasant thoughts. However, they also suggested that the MMORPG context could often provide a refuge from real-world issues, where new friendships and cooperative play could provide enjoyment. Correspondingly, a review of videogame use and flourishing mental health employing Seligman's 2011 positive psychology model of well-being (i.e., positive emotion; engagement; relationships; meaning and purpose; and accomplishment) reported that moderate levels of play was associated with improved mood and emotional regulation, decreased stress and emotional distress, and relaxation. Decisively, Jones and colleagues (Jones et al., 2014) asserted that “videogame research must move beyond a “good-bad” dichotomy and develop a more nuanced understanding about videogame play” (p. 7).

Despite the progress made, no systematic literature to date has synthesized the state of the empirical evidence considering the well-being influences of MMOs. This is important for three reasons: (a) MMOs have had significant advancements in the last 5 years, which may have radically altered their well-being potential (i.e., audio, visual, and augmented reality effects; Alha et al., 2019; Semanová, 2020); (b) the MMO players community has significantly expanded (Statista, 2021) and; (c) growing empirical evidence has widened the available knowledge of the effects of multiplayer gaming (Sourmelis et al., 2017; Cole et al., 2020). Consequently, this present systematic review will contribute to the niche research area referring to the MMOs and well-being association. To address this purpose, the notion of psychosocial well-being and its operationalization needs to be clarified. Scott and Porter-Armstrong (2013) conceived one's level of well-being as expressed through an individual's interpersonal and intrapersonal functioning. In that context, the complexity related to the assessment of one's well-being is acknowledged (Burns, 2015; Linton et al., 2016). On that basis, this review utilized the six broad well-being themes as delineated by Linton et al. (2016) to inform the theoretical framework of synthesizing MMO well-being related effects and evidence. The six themes are: (a) mental well-being (e.g., a person's thoughts and emotions); (b) social well-being (e.g., interactions and relationships with others, social support); (c) activities and functioning (e.g., daily activities and behavior); (d) physical well-being (e.g., person's physical functioning and capacity); (e) spiritual well-being (e.g., connection to something greater, faith) and; (f) personal circumstances (e.g., environmental factors; Linton et al., 2016).

To enhance the utility of findings, the present review will focus on the most prevalent age range of MMO gamers. The entertainment software association reported that of those playing video games, 21% are under the age of 18 years, 38% between 18 and 34, 26% between 35 and 54 and 15% 55 and over (Pierre-Louis, 2020). In addition, the currently most popular MMOs were identified and targeted. According to the entertainment software association, these involve World of Warcraft, RuneScape, and Guild Wars 2 among gamers older than 13 years (BeStreamer, 2020; Entertainment Software Association, 2020). All the available empirical evidence derived by randomized, controlled trials, cross-sectional studies, and case studies with *n* > 1 that identified any MMOs linked well-being outcomes was included and examined across the six well-being domains identified (see Linton et al., 2016). Thus, all the range of interpersonal and intrapersonal well-being outcomes for MMO players over the age of 13 were considered. The ultimate aim of this review is to contribute to balancing the available knowledge surrounding the impact of the popular MMO genre, whilst concurrently illustrating directions for gamer-centered and beneficial future research and mental health practice initiatives.

## Materials and Methods

This systematic review followed the methodology suggested in the Preferred Reporting Items for Systematic Reviews and Meta-analysis (PRISMA; Moher et al., 2009; Shamseer et al., 2015). Research team discussion and perusal of related published reviews assisted the development of the initial research eligibility, search strategy, and related terms. Inclusion and exclusion criteria were further refined at the selection process stage, after exposure and familiarity with the research area; this review was limited to research obtained from database searches.

### Eligibility Criteria

All research investigating massively multiplayer online gaming were eligible for review. The initial search eligibility criteria were (i) a publication date between 2012 to 2020; (ii) written in or translated into English language; and (iii) full-text, peer-reviewed primary research.

### Information Sources and Search Strategy

**Figure 1:**
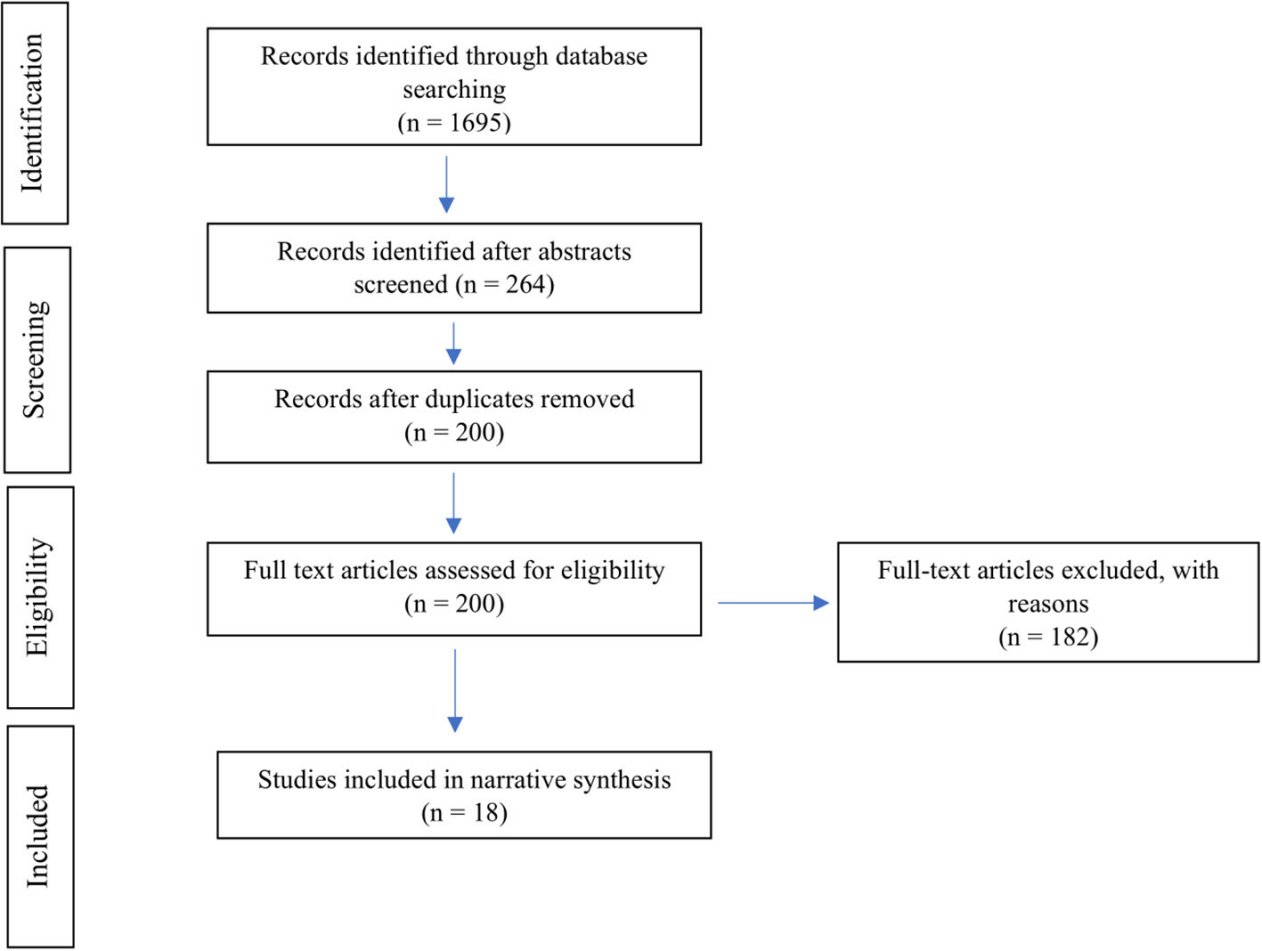
PRISMA flow diagram for the present study.

Searches were conducted in August 2020 using online databases, JB searched PsycNET (APA), and PUBMED; whereas, LR searched Scopus (see Figure 1). In each case, the following search terms and protocol were used (massively multiplayer online OR multiplayer online OR MMORPG OR MMOG) to search abstracts and/or titles. Searches were limited by publication date, 2012 to the present. No specific terms for well-being outcomes were prescribed to ensure that the literature search remained expansive. Accordingly, potential well-being effects were assessed at the screening stage.

### Selection Process and Data Management

After the title search, abstracts were independently screened by two investigators (JB & LR) for positive outcome measures, fitting within the identified well-being parameters (i.e., Linton et al., 2016). Example terms included, but were not limited to, “well-being,” “quality of life,” “social support,” “belonging,” “positive affect,” and “cognitive ability.” Where abstracts contained insufficient/unclear information, the full-text was reviewed for accurate evaluation. The resultant items/studies/records were pooled, and duplicates were removed. The remaining, potentially relevant studies were divided equally between LR and JB, and the full studies were subsequently (and independently) assessed. Where uncertainty of inclusion was noted, articles were screened by the alternate investigator (i.e., JB or LR). Then, if uncertainty regarding inclusion still remained, investigator LK was the final arbitrator (see Figure 1).

This detailed screening process utilized the following inclusion criteria: (i) qualitative or quantitative research of any design; (ii) written in or translated into English language; (iii) a primary study aim was psychological well-being (or a component of psychological well-being; Linton et al., 2016); and (iv) it was clearly indicated that participants were aged 13 years or over [according to Entertainment Software Association (2020) age ranges of high gaming prevalence]. Studies were excluded if: (i) they were single case studies, reviews of any kind (e.g., systematic reviews or meta-analyses), dissertations or theses, or opinions or discussion papers; (ii) the focus was IGD, problematic gaming or addiction; (iii) they involved online gambling, sexual foci (e.g., cybersex), exergaming, or e-sports; (iv) the game was not generally available to the wider community or was an educational tool; (v) they focused on motivations for engaging in online gaming or on learning English language; or (vi) gaming was not played on computers. Once articles were pooled, each reviewer independently recorded the reasons for excluding the articles in a shared file.

### Data Extraction Process

The final studies were summarized according to the following characteristics: (1) study design (e.g., cross-sectional survey); (2) sample characteristics (i.e., size, source of recruitment); (3) the specific MMORPG(s) emphasized; (4) variables (i.e., types of social capital, types of networks); (5) instruments for assessing key variables (e.g., time in game, social capital); (6) the type of analysis used; (7) main findings in relation to well-being (e.g., relationship between game and well-being or with belongingness); and (8) limitations. Investigators SR and LR each independently reviewed half of the studies, with joint discussion to resolve any uncertainties. Table 1 summarizes the reviewed studies.

**Table 1:** Main characteristics of reviewed studies (*N* = 18).

**Study ID**	**References**	**Sample characteristics**	**Game/s**	**Design**	**Aim and purpose**	**Well-Being measure**	**Findings/Results**
1	Ahlstrom et al. (2012)	*N* = 349 Heterosexual Couples	WoW, lord of the rings, eve online, final fantasy zi, guild wars, city of heroes and other MMORPGs	Cross-sectional	To identify differences in marital satisfaction for couples who game together and couples where only one partner games	Revised dyadic adjustment scale–marital satisfaction	Compared to 1 gamer couples, couples gaming together = greater marital satisfaction. Where only 1 partner games, can lead to lower marital satisfaction due to immersion in the game–like any leisure activity
2	Castillo (2019)	*N* = 147	World of warcraft	Cross-sectional	To examine the differential effects of social and individualistic motivations to play MMO games on feelings of general social support and tolerance	Internet social capital scales (ISCS)−4 items	Players who want to form relationships are more likely to experience greater bridging social capital
3	Choi (2019)	*N* = 228	Not identified (MMORPG)	Cross-sectional	Does avatar identification affect self-esteem?	Social interaction, Berlin social support scale, avatar self-identification, ISCS and interpersonal support assessment list, Rosenburg self-esteem scale (SES)	Avatar identification increases social capital and self-esteem in real lives. Social interaction in MMORPG increases social capital in real lives through avatar self-identification. Social support perceived in MMORPGs increase self-esteem in real lives through avatar self-identification
4	Cole et al. (2020)	*N* = 337	WoW, final fantasy-XIV, or elder scrolls online	Cross-sectional	Is MMORPG play associated with healthy outcomes due to higher online social support and are adverse outcomes a function of online peer victimization?	Cyber victimization scale, online social support scale, perceived victimization in the workplace scale, the interpersonal support evaluation list, the beck depression inventory, SES, the perceived stress scale	Playing more can lead to greater social support and peer victimization. Increased social support leads to lower depressive and anxiety symptoms & increased self-esteem. Games may be a new source to receive social support
5	Doh and Whang (2014)	*N* = 37	Mabinogi	Q-methodology	To understand the identity development of adult players in an online world	Behavioral statements representing identity development in the gaming world	When players have different motivations to play–they have different meaning outcomes. Games can provide a behavioral setting in which to experience different paths of identity development
6	Gallup et al. (2016)	*N* = 3 ASD diagnosis	Not identified (MMORPG)	Qualitative	To explore whether online gaming can help young people with ASD develop communication and relationship skills and improve post-secondary education	Open, in-depth interviews about the meanings of social interactions	The online game provided a place for these participants to have social connections and relationships compared to difficult off-line relationships. 4 themes found including generalizing skills to the real world
7	Gallup et al. (2017)	*N* = 5 ASD diagnosis	Not identified (MMORPG)	Qualitative	To identify the potential of using the online gaming environment to support the development of social skills	Semi-structured interviews and in-game observations of social interactions	MMORPGs help connect individuals with ASD to a community, develop communication and relationships skills and identify supports that will improve postsecondary transition and increase persistence in postsecondary education
8	Kaye et al. (2017)	*N* = 708	Not identified (MMO)	Cross-sectional	To examine the role of gamer identity and online social capital as mediators of online gaming engagement and psychosocial outcomes	Group identification scale, ISCS, SES, UCLA Loneliness scale, CPI:SY–subscale of the IPIP Scale	There was a positive relationship between MMO engagement, gamer identity and online social capital. Gamer identity was positive correlated with self-esteem and social competence and negatively correlated with loneliness
9	Martončik and Lokša (2016)	*N* = 161	World of warcraft	Cross-sectional	Do WoW players experience less loneliness and social anxiety in the online world than in the real world?	UCLA loneliness scale, social phobia inventory	People perceived their level of loneliness and anxiety as significantly lower in online world than in the real world. Playing with known others = decreased loneliness in real world
10	Meng et al. (2015)	*N* = 17,995	League of legends	Cross-sectional	Does multiplayer online battle arena game play and the types of connections made, influence social capital?	ISCS	Multi-modal connectedness is associated with bonding and bridging social capital. Frequency of play with existing offline friends was positively correlated with both bridging and bonding social capital. Play frequency with new online friends was positively correlated with bridging social capital
11	O'Connor et al. (2015)	*N* = 22	World of warcraft	Qualitative	Do MMOG players receive social support for emotional and other offline issues, online?	Questions measuring psychological sense of community, social identity, social support	Participants reported experiencing an MMOG-based sense of community and received social support from others within the gaming community
12	Perry et al. (2018)	*N* = 2030	Destiny	Cross-sectional	To examine the associations between time spent playing with other people on bridging and bonding social capital and indirect associations via harmonious and obsessive passion	10 item version of Vallerand's Passion Scale, ISCS	Harmonious but not obsessive passion would mediate the positive association between playing with others and social capital. Real-life friends were positively associated with bonding social capital, strangers with bridging social capital, and online-only friends with both
13	Shen and Chen (2015)	*N* = 18,813	Chinese chevaliers romance 3	Cross-sectional	To examine the relationship between social capital, co-playing patterns and health disruptions	Health disruption question, internet social capital scales, general social survey	Bonding social capital reduced risk of health disruption. Playing with friends first met in the game reduced health disruption–while playing with family and friends increased health disruption
14	Voulgari et al. (2014)	*N* = 27 (qual) *N* = 221 (min)	WoW, lineage II, ikariam, lord of the rings online, EVE online, aion, tribal wars, league of legends, darkfall, age of conan, guild wars, city of heroes/villains final fantasy XI	Mixed method	To explore the learning outcomes and processes emerging in the environment of MMOGs	Learning outcomes, cognitive outcomes, skill based outcomes, social skills, affective impact	Playing games had positive impacts on gaining social skills, cognitive skills, skill acquisition and had affective impact. Acquisition of cognitive and social skills transferred to real life
15	Xanthopoulou and Papagiannidis (2012)	1st Measurement *N* = 299 2nd Measurement *N* = 79 Employed	WoW Lineage II Lord of the rings online EVE online Second life	Longitudinal	To examine how participating in massively multiplayer online role-playing games affects users' real-life employment	4-item mastery subscale of the recovery experience questionnaire, multi-factor leadership questionnaire	In-game active learning and transformational leadership can spill over into work life when there is enhanced game performance
16	Zhang and Kaufman (2015)	*N* = 222 over age 55	World of warcraft	Cross-sectional	To analyse the relationships between older adults' social interactions in MMORPGs and their online social capital	ISCS	Higher levels of bridging and bonding social capital for older adults are dependent on the contexts of game play and enjoyment of relationships
17	Zhang and Kaufman (2016)	*N* = 354 Age over 55	World of warcraft	Cross-sectional	To investigate older adults' social interactions in massively multiplayer online role-playing games (MMORPGs)	Social interactions within WoW, Liu and Peng's (2008) study of problematic Internet use	Playing MMORPGs offered older adults' opportunities to sustain off-line relationships with family and real-life friends and to build meaningful and supportive relationships with game friends
18	Zhang and Kaufman (2017)	*N* = 222 Age over 55	World of warcraft	Cross-sectional	To explore the degree to which older adults' social interactions in massively multiplayer online role-playing games (MMORPGs) are associated with four socio-emotional factors	Short-form UCLA Loneliness scale, center for epidemiological studies depression scale, multidimensional scale of perceived social support, social connectedness scale	Older adults' socio-emotional well-being was associated with the quality of guild play and enjoyment of relationship. The findings suggest that the relationships made with online friends wouldn't easily integrate into their off-line lives

### Data Analysis Procedures and Quality

Given the diversity of study objectives and well-being outcomes reviewed, meta-analysis was not plausible. Therefore, a narrative synthesis methodology was adopted, as it involves a textual summation and explanation of the data which was considered appropriate considering the focus of this review (Greenhalgh et al., 2005; Popay et al., 2006). Following the goals of this review, the analysis aimed to identify the key positive or well-being outcomes of playing MMORPGs. Consequently, comparable studies/results were grouped together categorizing the data into themes (and subthemes) that drew on the six well-being themes identified by Linton et al. (2016). A narrative account of these results is presented under relevant thematic headings, along with any pertinent moderating factors (Greenhalgh et al., 2005).

Risk of bias and quality of evidence evaluations were undertaken using the Appraisal tool for Cross-Sectional Studies (Downes et al., 2016) for the quantitative studies, and the Critical Appraisal Checklist for Qualitative Research (Joanna Briggs Institute, 2020) for the studies that used a qualitative methodology. The Mixed Methods Appraisal Tool (Hong et al., 2018) was used by JB and LR to conduct their independent appraisals of each study. These were then compared and discussed across each item/study/record to conclude agreement.

## Results

### Study Selection

As per the flow of information and studies is shown in Figure 1, a total of 1695 studies (PsycNET *n* = 524, PubMed *n* = 500, Scopus *n* = 671) were identified through the initial search. After abstracts were reviewed, 1,431 studies were excluded due to not being suitable for the present review. A further 64 studies were removed for duplication. A full-text review was done on the remaining 200 studies. Of these 182 studies were excluded due to age of participants (*n* = 8), focus on IGD or addiction (*n* = 32), focus on motivations/predictors of play (*n* = 24), not being in English (*n* = 4), not being primary research (*n* = 30), focused on education (*n* = 16), full-text unable to be accessed (*n* = 4), not exclusively MMO (*n* = 8), only measuring in-game behaviors (*n* = 29), or not meeting well-being criteria (*n* = 27). Following this screening process, 18 studies were included in the final narrative synthesis (see Figure 1).

### Study Characteristics

The main characteristics, including the aims and purpose of each study, the well-being measures used, and the results of each of the final 18 studies are noted Table 1. For those studies which reported the gender of their participants, males accounted for the majority, ranging from 65 to 100% [the latter being the case in the qualitative study of Gallup et al. (2016)]. One study was equally represented gender-wise (Cole et al., 2020) and one had slightly more females (51%) than males (Doh and Whang, 2014). Participants were from North America, China, Korea, Greece, and Australia. For those studies that reported the game platform, World of Warcraft was the most common (*n* = 10). Twelve studies measured time spent gaming with variable time measures, such as hours weekly, per week-day, and weekend. Averages of hours per week ranged from 11 to 36.7, while daily hours were estimated to vary between 2 and 5.

### Risk of Bias and Quality of Studies

Quality of reporting, study design quality and risk of bias was assessed for each of the 13 cross-sectional studies. All the cross-sectional studies had a moderate level of risk of bias [studies: 1–4, 8–10, 12, 13, 15-18]. This included sample issues [studies, 1-4, 8-10, 12, 13, 15, 17, 18]. Only one study provided information to justify their sample size, and this was through pragmatic rather than statistical reasons (Zhang and Kaufman, 2015). Although seven studies [studies, 1, 4, 8, 10, 12, 13, 17] had sample sizes over 300, sample size was deemed to be an issue of concern given the millions of MMOG players globally (Internet World Stats, 2020). Sampling methods raised concerns regarding risk of bias and study design quality, as most studies relied on self-selection, and one MMOG was the primary data collection source [six studies used this MMOG alone (studies 2, 9, 11, 16–18), while four studies (studies 1, 4, 14, 15) included this MMOG], although conclusions were often made with reference to MMOGs as a whole. Only six studies [studies, 2, 3, 10, 13, 15, 16] acknowledged or raised concerns regarding response rates, but did not provide clear information on this or expected response rates due to the impossibility of determining sampling frames. Furthermore, due to participant self-selection, the majority of studies did not compare responders and non-responders. Of the two studies [4, 15] that did consider response bias, one (Cole et al., 2020) found no difference between non-completers and completers, while the other (Xanthopoulou and Papagiannidis, 2012) found differences on four demographic characteristics (age, gender, occupational, and marital status). Considering the quality of design, the majority of the 13 cross-sectional studies were deemed to fall into a fair category, with a major concern being the omission of whether ethical approval or participant consent was obtained [studies 2, 3, 8–10, 12, 13, 15] and only three studies reporting that there were no funding or other conflicts [studies 2, 12, 17].

The Joanna Briggs Institute (JBI) critical appraisal checklist for qualitative research was used to assess risk of bias for the qualitative studies (Joanna Briggs Institute, 2020). Overall, the quality of these four studies [5, 6, 7, 11] was assessed as quite good. The JBI checklist highlighted two key concerns: adequate reporting of the positioning and of the research influence of the investigators. Only two of the four studies provided details as to the role or possible influence of the investigators on the research [studies 5, 7], and only one study [7] provided a statement showing the cultural and or theoretical perspective of the investigator.

### Outcomes

Of the 18 studies, four were qualitative [5, 6, 7, 11] one was a mixed method design [14] and the others were all cross-sectional by design [1–4, 8–10, 12, 13, 15–18]. This led to all results showing exclusively correlational and/or regression links/effects, with unclear direction of causality regarding the MMO gaming and well-being experiences association. Only one study (Xanthopoulou and Papagiannidis, 2012) was longitudinal in design with the second measurement being obtained 1 month after the first responses were collected, allowing for stronger predictive inference.

The well-being outcomes assessed in all the studies were operationalized similarly to authors' expectations aligning with the framework provided by Linton et al. (2016). Two predominant types of positive outcomes were addressed by the included studies: social well-being and mental well-being. Additionally, one study (Shen and Chen, 2015) [13] considered physical well-being. Several game attributes were considered as predictors across the studies reviewed. The most common attribute was the social aspect as examined by 15 studies [2–4, 6–14, 16–18]. This referred to modes of communication (e.g., in-game talk, game bulletin boards, online comms outside the game), “who” the gamers play with (e.g., real-world friends, on-line friends, family), and time spent gaming. The synthesized results are presented through the lenses of the 2 main well-being outcomes identified.

#### Social Well-Being

Of the 18 studies, 15 included some form of measurement of social well-being. O'Connor et al. (2015) [study 11] reported that participants of WoW game received social support from others within this gaming community. Gallup et al. (2016) [study 6] and Gallup et al. (2017) [study 7] found that using the online game environment was beneficial for secondary and tertiary students with an Autism Spectrum Disorder (ASD) diagnosis, to develop social connections as well as communication and relationship skills. This skill development also led to improved post-secondary education transitioning. Cole et al. (2020) [study 4] also looked at whether social support increased in the gaming environment, finding that more time spent in playing in guilds as related to higher levels of social support, and that this was correlated with cognitive-emotional outcomes. Additionally, they compared on-line and in-person social support and outcomes, finding differential effects. Cole et al. (2020) [study 4] concluded that MMOGs represent different social support environments, and as such, online worlds could be used as a new and different source of social support. These findings are echoed by Voulgari et al. (2014) [study 14], whose mixed methods research across more than 10 MMOGs found that gaming developed collaborative skills and social bonds additional to real-life relationships. Moreover, gaming constituted a part of the gamers' existing real-world social life.

Social capital effects investigated by the reviewed studies included bonding and bridging aspects. Bonding related social capital implies a deeper form of social support, experienced by those with whom one maintains emotional intimacy, such as their family and friends (Meng et al., 2015) [study 10]. In the game context, bonding social capital refers to the support networks within a specific online gaming group or community, such as one's guild (i.e., group of in-game allies) or group within a particular game (Claridge, 2020). Bridging social capital refers to the support, mainly by sharing information and resources, one may experience from broader and less intimate social groups they belong into, such as their social class, race, and religion (Perry et al., 2018) [study 12]. Castillo (2019) [study 2] found greater bridging social capital experienced when gamers presented more motivated to form relationships with others, compared to gaming for competitive reasons. Moreover, Meng et al. (2015) [study 10] found that playing frequently in the online gaming environment with existing offline friends was positively correlated with both higher bridging and higher bonding social capital. This aligned with Kaye et al. (2017) findings, that playing with online and real-world friends, as well as online interactions in-game and outside, was positively related to both higher bridging and higher bonding social capital.

The study by Perry et al. (2018) [study 12] reported that harmonious passion for playing MMOGs helped build social capital; however, when this passion was obsessive, the outcomes were negative. Their study further found that playing with real-life friends was positively associated with higher bonding social capital experienced by gamers. Interestingly, playing with strangers, and possible new friends, was positively associated with increased bridging social capital. Choi (2019) [study 3] extended such findings by focusing on the link between a gamer's social interactions, avatar identification, and social capital. Higher avatar (i.e., in-game figure representing the gamer) identification was related to increased real-life social capital, with one's greater perception of in-game social interactions linked to higher levels of avatar identification and subsequently elevated social capital.

Three of the articles reviewed [Studies 16, 17, & 18] focused specifically on social well-being among older populations, with all participants exceeding 55 years. These studies by Zhang and Kaufman (2015) [study 16], Zhang and Kaufman (2016) [study 17], and Zhang and Kaufman (2017) [study 18] all looked at the social interactions of older adults in MMORPGs. It was found that enjoyment of relationships in the online game was positively related to both bridging and bonding social capital, and this was partly associated to a gamer's amount of game play, active participation in guilds, and their reported enjoyment of the game. The same three studies also suggested that gaming contributed to maintaining existing family and friend relationships, as well as the development of new meaningful friendships. One of the studies, did imply, however, that new online friends did not easily integrate into the older gamers' real lives (Zhang and Kaufman, 2017) [study 18]. They explained that as the result of older adults' lesser need for large networks, as well as geographical limitations.

Lastly, one article looked at social well-being through the lens of marital satisfaction (Ahlstrom et al., 2012) [study 1]. They reported that compared to couples where only one member is a gamer, couples who game together experience higher levels of marital satisfaction. Higher marital satisfaction was related to more time spent in in-game interaction and higher satisfaction of playing together. They supported that gaming is a leisure activity, where when only one person is immersed, disruption to marital harmony may be caused. Indeed, this was confirmed by both types of couples (e. g., only one gaming vs. both gaming), when considering their different or similar bedtimes and their arguments over the time spent in gaming compared to the time spent together.

#### Mental Well-Being

A smaller proportion of studies looked at the effects of MMOG on components of mental well-being such as self-esteem, depression, stress, general affect, and skill acquisition. Self-esteem was specifically identified in three articles [Studies 3, 4, & 8] and was related to social support received in the game and with positive gamer identities in an MMORPG (Kaye et al., 2017; Choi, 2019; Cole et al., 2020). In their study investigating MMO involvement, gamer identity, and social capital, Kaye et al. (2017) [study 8] found that higher MMO involvement increased with higher bonding and bridging social capital and solidified gamers' identity, which in turn increased their self-esteem and decreased their loneliness. Similarly, Choi's 2019 [study 3] study into the effects of avatar self-identification indicated that perceptions of social support from MMORPG increased avatar identification alongside the gamers' real-life self-esteem. In their examination of a Compensatory Social Interaction Model, Cole et al. (2020) [study 2] investigated the associations between one's MMORPG guild play, social support, peer victimization, self-esteem, depression and stress. Gamers who engaged more in guild play, experienced higher levels of social support (compared to levels of peer victimization), which resulted in improved self-esteem, lower depression, and stress symptoms. Martončik and Lokša (2016) [study 9] directly looked at the social effects of WoW's (i.e., guild affiliation, communication used) on individual's mental well-being. Their study revealed that gamers perceived their level of loneliness as significantly lower in the online world than in the real world. Additionally, gaming with others already known to the player in their real-life decreased perceptions of real-world loneliness. Martončik and Lokša (2016) [study 9] also found that levels of anxiety were lower in the online world, when gamers perceived themselves as less lonely. Similarly, lower levels of loneliness and depression among gamers aged over 55 years were predicted by higher quality of guild play [study 18]. This suggested that for older adults, being an active member of an in-game guild, may improve their emotional well-being (Zhang and Kaufman, 2017).

The mixed methods study by Voulgari et al. (2014) [study 14] contributed information across a combination of different social, cognitive, and emotional well-being outcomes of gaming. Their study found that playing MMOGs had positive impacts on gaining social skills and improving cognitive skills, as well as a positive affective impact. The cognitive skills they identified to have been improved included procedural knowledge and problem-solving skills. The acquisition of such cognitive and social skills was reported to be transferable into their offline world. The authors also reported that for some gamers, positive affective impacts, such as enjoyment and satisfaction, were the most important outcomes. In-game and work leadership skills were looked at by Xanthopoulou and Papagiannidis (2012) [study 15] in their examination on the effects of gaming on real-life employment. They found that in-game active learning was reflected in active learning at work, but only for high game performers. Moreover, transformational leadership was shown to spill over into a player's work life, although this appears to be enhanced by higher game performance.

In that line, Doh and Whang (2014) focused on the development of behavioral statements to establish the gaming environment as a different pathway to use in identity development. They reported that a player's motivation to participate in online gaming could progressively lead to an alternated identity. Lastly, Shen and Chen (2015) explored the effect of gaming related social capital into health-related outcomes. This study found that bonding and not bridging social capital occurring while playing online related to reduced health disruption in one's daily lives.

## Discussion

The increasing preference for MMO gaming for leisure and e-sport has led to a large body of research investigating the possible adverse outcomes related to their excessive usage (Stavropoulos et al., 2019, 2020). However, less is known about the possible benefits of moderate MMO gaming for one's individual psychosocial well-being. The aim of this review was two-fold: (a) to identify and summarize the empirical evidence for the potential interpersonal and intrapersonal positive well-being outcomes for non-excessive MMO players over the age of 13; and (b) to identify possible research priorities in relation to better understanding the beneficial effects of MMO gaming. Overall, a positive relationship between playing MMOs and social well-being was found.

This systematic review identified 18 studies that were published between 2012 and 2020, and which investigated the adaptive well-being outcomes of MMOG for adolescent and adult players. These studies examined two key aspects of psychosocial well-being, as defined by Linton et al. (2016). Firstly, one's social well-being, encompassing individuals' connections with others—their interactions, their depth of relationships, and the social support their connections provided, was emphasized by the reviewed empirical evidence. This was the dominant topic of interest, while the gamers' mental well-being (e.g., individual psychological, emotional, and cognitive aspects) followed. In order to investigate these outcomes, gaming attributes such as gaming time, game performance, gamer identity, types of communication one is engaged in, type of co-players (e.g., online or offline friends, family, strangers), and guild membership were examined.

In that context, a commonly used measure of social well-being employed in the studies reviewed was social capital. The significant positive relationship found between MMOG engagement and bridging and bonding social capital in those studies appears promising. Specifically, reviewed findings in studies 2, 10, 12, and 16 suggest there is strong support for the notion that MMO gaming may foster one's social well-being in both virtual worlds and in their off-line lives (Meng et al., 2015; Zhang and Kaufman, 2015; Perry et al., 2018; Castillo, 2019). Moreover, such evidence is strengthened by studies 1, 3, 4, 6, & 18, which utilized more discrete measures of social well-being, such as one's perceptions of social support, social interactions, and marital satisfaction, showing that MMO gaming bolstered these too (Ahlstrom et al., 2012; Gallup et al., 2016; Zhang and Kaufman, 2017; Choi, 2019; Cole et al., 2020). These overall positive conclusive impacts on one's social well-being seem to be reasonably robust given (a) the diverse game attributes considered in these studies (e.g., time spent in play, gamer identity, frequency of play with different types of co-players, avatar identification); and (b) the diverse age and ethnicities of gamers that these impacts were found with-including a small and unique group of gamers with ASD. Moreover, the impacts of MMORPG on social well-being were apparent in both quantitative and qualitative research. Nevertheless, and in line with the current PRISMA systematic literature review's study eligibility criteria, it should be reiterated that the majority of the gamers in the studies reviewed were classified as non-problematic gamers, with study 5 actively excluding those who fit criteria for addiction (e.g., Doh and Whang, 2014). Similarly, reviewed studies 12 and 18 included gamers who could be classified as experienced and/or as heavy users, yet they had received no formal diagnosis (Zhang and Kaufman, 2017; Perry et al., 2018). Thus, due to the wide range of time participants spent gaming, the findings are applicable to both the more casual and immersed gamer populations, solidifying the positive effects of MMO gaming on one's social well-being.

Further, the reviewed studies examined the mental well-being effects of one's MMO gaming. Self-esteem, loneliness, depression, and positive affect were the main psychological outcomes investigated, while studies 7 and 14 looked at cognitive skill acquisition (Voulgari et al., 2014; Gallup et al., 2017). Overall, these studies found that gaming bolstered self-esteem, and reduced depression, stress, and loneliness, whilst fostering cognitive and social skills. However, these positive findings should be treated with some caution, as these variables were only considered in a handful of the studies and such revealed effects may be interwoven with one's concurrently experienced positive social well-being outcomes. More studies need to be conducted among MMO gamers, in which mental well-being outcomes are of primary focus, and social variables are controlled for.

Taken together, this review provides validation to game developers, educators, health professionals, and policy makers, that despite evidence regarding the adverse outcomes of excessive MMO gaming and problematic gaming behavior, there are important psychosocial benefits to be gained from moderate and adaptive gaming. This information is relevant to game developers as they should be encouraged to find ways to enhance social contact opportunities. Moreover, it is important that health professionals and educators are aware that MMO gaming is an avenue for social connection and support, similar to other real-world leisure and sporting pursuits. Pathologizing gaming could well undermine the identity, social, and psychological well-being of those who actively benefit by their moderate and adaptive gaming engagement.

### Strengths and Limitations

The validity of these results is restricted due to the heterogeneity of methodologies used in the studies reviewed. Although qualitative and quantitative empirical evidence was included, most studies used a descriptive design to assess the self-reported effects of MMO gaming on well-being. Moreover, although many of the studies controlled for some covariates, such as demographic variables or gaming time, variables of interest were narrow, and other unmeasured variables might account for some of the observed effects. Additionally, although many of the predictor measures had solid theoretical bases, others have not been fully trialed (e.g., intensity of interaction, multimodal connectedness), contributing to possible validity issues. Furthermore, the value of the findings is impacted by a lack of generalizable results. For example, self-selection bias was reported by several studies, where heavy gamers or an overly well-educated sample was used, and some studies looked at specific populations (e.g., 55+ years, those with ASD; Zhang and Kaufman, 2015; Gallup et al., 2017) [See studies 7 & 16]. The sample of MMO games examined was also narrow, with WoW dominating. Finally, only a limited number of well-being constructs were examined by the 18 studies, thus the conclusions regarding well-being have limited generalizability/need to be treated with caution due to narrow constructs covered. Of note was a lack of variety in the well-being outcomes being studied. While social well-being is an important part of MMO gaming, little is known about other aspects of well-being such as mental well-being, spiritual well-being, and physical well-being. The fact that no randomized control trials have been undertaken to contribute to the research on well-being outcomes and MMO participation is an important omission in this field of study.

This review was limited to peer-reviewed studies published in three academic databases between 2012 and August 2020, at one particular point in time. Therefore, the review may be subject to English-language and publication bias, and the studies included may not be a representative sample. Relevant research may also have been missed due to including the use of selected search terms, and this review did not include non-peer-reviewed literature (e.g., theses, conference proceedings), which may have omitted important data. Finally, well-being is a broad concept, and other reviews may generate different empirical evidence dependent on the operationalizations followed.

Despite the noted review-level limitations, this study has several strengths. First, this review used rigorous methodology, following PRISMA guidelines and assessing quality and risk of bias using validated tools. Additionally, the inclusivity of study design has meant we have captured data through diverse approaches with similar outcomes. Finally, the broad search parameters with regards well-being ensured that we did not limit the construct to narrow conceptualizations of well-being outcomes related to MMO gaming.

### Conclusion

This review has offered a valuable examination of the current research on the psychosocial benefits of multiplayer online gaming. It is important to note the number of reviewed studies that reported significant positive outcomes regarding social well-being. The major limitation of the review relates to the modest quality of research in the area, and the limited aspects of well-being investigated to date. While social well-being is an important part of MMO gaming, there is very little known about other aspects of well-being such as mental well-being, spiritual well-being, and physical well-being.

Recommendations for future research include broadening the well-being constructs that are investigated in relation to gaming. Clear and consistent operationalization of commonly used variables and measures and standardized demographic information would provide greater validity and comparability of results. Longitudinal research in which baseline measurements of well-being and other variables are taken to assess changes in this outcome, to determine causation and not merely correlational effects is also required. Finally, using a greater variety of gaming platforms, instead of mostly WoW, would provide increased robustness for positive well-being outcomes related to MMOGs.

## Data Availability Statement

The original contributions presented in the study are included in the article, further inquiries can be directed to the corresponding author/s.

## Author Contributions

LR and JB performed the bibliographic search, participated in the selection of included studies, resolved methodological doubts of possible studies, and helped in the all versions of this manuscript. LK-D and VS were senior authors and were involved in the review design and review aim, also the above processes conducted by LR and JB, and manuscript revision and submission. PM, AA, HS, JM, TD, and AW contributed in the interpretation of the results and the improvement of the manuscript. PM also contributed to mentoring in the PRISMA process. All authors contributed to the article and approved the submitted version.

## Conflict of Interest

The authors declare that the research was conducted in the absence of any commercial or financial relationships that could be construed as a potential conflict of interest.
